# The role of TGFβ1 and LRG1 in cardiac remodelling and heart failure

**DOI:** 10.1007/s12551-014-0158-y

**Published:** 2015-01-15

**Authors:** Weihua Song, Xiaomeng Wang

**Affiliations:** 1 Division of Metabolic Medicine, Lee Kong Chian School of Medicine, Nanyang Technological University, Research Techno Plaza, X-Frontiers Block, Level 4, 50 Nan yang Drive, Singapore, 637553 Singapore; 2 Division of Cell Biology in Health and Disease, Institute of Molecular and Cell Biology, Singapore Agency for Science, Technology and Research, 61 Biopolis Drive, Proteos, Singapore, 138673 Singapore; 3 Department of Cell Biology, Institute of Ophthalmology, University College London, 11-43 Bath Street, London, EC1V 9EL UK

**Keywords:** LRG1, TGFβ, Cardiac remodelling, Therapeutic angiogenesis, Fibrosis, Heart failure

## Abstract

Heart failure is a life-threatening condition that carries a considerable emotional and socio-economic burden. As a result of the global increase in the ageing population, sedentary life-style, increased prevalence of risk factors, and improved survival from cardiovascular events, the incidence of heart failure will continue to rise. Despite the advances in current cardiovascular therapies, many patients are not suitable for or may not benefit from conventional treatments. Thus, more effective therapies are required. Transforming growth factor (TGF) β family of cytokines is involved in heart development and dys-regulated TGFβ signalling is commonly associated with fibrosis, aberrant angiogenesis and accelerated progression into heart failure. Therefore, a potential therapeutic pathway is to modulate TGFβ signalling; however, broad blockage of TGFβ signalling may cause unwanted side effects due to its pivotal role in tissue homeostasis. We found that leucine-rich α-2 glycoprotein 1 (LRG1) promotes blood vessel formation via regulating the context-dependent endothelial TGFβ signalling. This review will focus on the interaction between LRG1 and TGFβ signalling, their involvement in the pathogenesis of heart failure, and the potential for LRG1 to function as a novel therapeutic target.

## Introduction

Heart failure is a progressive and chronic condition in which the heart is no longer able to circulate blood efficiently to meet the body’s demands (Johnson 2014). A wide range of conditions such as ischemic heart disease (IHD), hypertension, valvular heart disease, myocarditis, diabetes and cardiomyopathy can lead to heart failure (Nishimura et al. 2014). In response to stress or injury, the myocardium undergoes a series of pathological changes including structural rearrangement and morphological changes of cardiomyocytes, inflammation, extracellular matrix (ECM) remodelling, microvascular rarefaction and chamber dilation (Manabe et al. 2002; Kehat and Molkentin 2010). These changes cause further deterioration in cardiac function and eventually lead to heart failure (Cohn et al. 2000).

Over the last few decades, the prevalence and incidence of heart failure continues to rise mainly due to the prolonged longevity, improved survival rate from other cardiovascular events (e.g., myocardial infarction, valvular disease, and arrhythmias), sedentary life style, and the increased prevalence of risk factors (e.g., hypertension, diabetes and obesity) (Mann DL 2012). In 2010, more than 41 million people lived with heart failure worldwide (Forouzanfar et al. 2013). Despite improved understanding of the molecular mechanisms and significant advances in treatment strategies, heart failure still carries substantial morbidity and mortality and its therapy remains a major unmet medical need. In this review, we summarise the current knowledge on the role of TGFβ1 and its novel modulator, LRG1, in different pathologies of cardiac remodelling and the potential of LRG1-targeted therapeutics for the treatment of heart failure.

## The challenges of current treatments for heart failure

Heart failure is a heterogeneous disease with a broad spectrum of symptoms. Current treatments aim to alleviate symptoms, slow disease progression and thereby improve overall quality of life and survival. For example, IHD-induced heart failure, the most common type of the disease, is normally treated with antiplatelet drugs, anticoagulants and β-blockers. Emergent reperfusion via surgical or catheter-based revascularisation procedures is used to restore blood flow and improve survival following ischemic episodes (Heuser et al. 2000; Horvath 2000). However, a substantial portion of patients are not suitable for or do not benefit from conventional revascularisation treatments because of a poor overall health status or the presence of comorbidities (Norgren et al. 2007). Even in patients who received successful primary revascularisation, stent thrombosis and saphenous vein bypass graft disease can occur and cause recurrent myocardial ischemia and cardiac remodelling (Kaul et al. 1991). Furthermore, none of these treatments alters the natural history of heart failure and therefore offers no cure.

Both human and animal studies have shown that individuals with robust collateral circulation and microvascular perfusion are associated with delayed myocardial cell death (Antoniucci et al. 2002), reduced occurrence of myocardial infarction (MI) (Choi et al. 2013), smaller infarction size (Habib et al. 1991) and increased survival (Meier et al. 2012). However, the capacity of collateral and capillary vessel remodelling under ischemic condition is highly variable among individuals. Accelerating this innate physiological response by exogenous angiogenic factors has been considered as an attractive approach to bypass occluded vessels, revascularise ischemic tissues and restore tissue function (Carmeliet and Jain 2011). An impressive body of pre-clinical evidence has demonstrated improved myocardial perfusion and function upon therapeutic angiogenesis in animal models (Harada et al. 1994; Unger et al. 1994; Landau et al. 1995; Lazarous et al. 1996; Shou et al. 1997; Lopez et al. 1998; Lee et al. 2000; Zhang et al. 2002; Cao et al. 2005; Heinl-Green et al. 2005; Cao 2009). Initial phase I clinical trials in patients with advanced IHD, but who did not meet the criteria for standard revascularisation strategies, have also demonstrated an improved cardiac circulation and function after being treated with pro-angiogenic factors such as vascular endothelial growth factors (VEGF) and basic fibroblast growth factor (bFGF) (Losordo et al. 1998, 2002; Schumacher et al. 1998; Rosengart et al. 1999; Symes et al. 1999; Hendel et al. 2000; Udelson et al. 2000; Henry et al. 2001; Vale et al. 2001; Fortuin et al. 2003; Reilly et al. 2005). However, caution is needed in the interpretation of outcomes of these studies as most of them lack proper placebo controls. Not surprisingly, similar therapeutic efficacy has not yet been achieved in larger, placebo-controlled, late-stage clinical trials, which is partly due to the extent of angiogenesis observed in the placebo group (Grines et al. 2002, 2003; Simons et al. 2002; Kastrup et al. 2011).

Angiogenesis is a tightly controlled process involving multiple levels of interactions between a wide variety of molecules, cells and extracellular matrix (ECM) proteins. It is now widely accepted that a single angiogenic factor may not be sufficient to induce the formation of functional vasculatures. Indeed, the treatment of VEGF leads to the formation of leaky, chaotic and tortuous vessels that lack the normal hierarchical structure (Nagy et al. 2007; Hedlund et al. 2009; Cao et al. 2010). Furthermore, both VEGF (Thurston 2002) and bFGF (Cuevas et al. 1991) are involved in vessel dilation and their treatment is associated with severe hypotension (Hariawala et al. 1996; Horowitz et al. 1997; Unger et al. 2000; Henry et al. 2001). In addition, there is evidence that VEGF exerts detrimental pro-atherogenic effects by influencing endothelial and immune cell function (Ross 1993; Inoue et al. 1998; Kim et al. 2001). A combination treatment targeting growth factors with complementary mechanisms might be more effective and has less unwanted side effects.

ECM is essential for proper cardiac function. It provides a scaffold for different types of cells in myocardium and transmits mechanical force and signals to myocardial fibres (Banerjee et al. 2006). ECM remodelling is a critical step that allows the ordered replacement of damaged cells after injury. However, chronic inflammation and repetitive injury can cause disturbed ECM homeostasis and fibrosis, a feature shared by many conditions associated with heart failure (Weber et al. 1995). Cardiac fibrosis exaggerates mechanical stiffness of the myocardium and its vasculature, impairs myocyte contractility, disrupts electrical coupling, destroys normal tissue architecture and eventually leads to heart failure (Lopez et al. 2001; Ho et al. 2010; Karagueuzian 2011). Increasing evidence shows that fibrosis is a dynamic and reversible process (Iredale 2007). Targeting fibrosis, therefore, presents a promising strategy to prevent or slow down the deterioration of cardiac function. Despite its huge impact on cardiovascular diseases and intensive research efforts to explore new therapies, there is no approved treatment that directly targets the mechanisms of fibrosis in the heart.

## TGFβ1 and heart failure

**Fig. 1:**
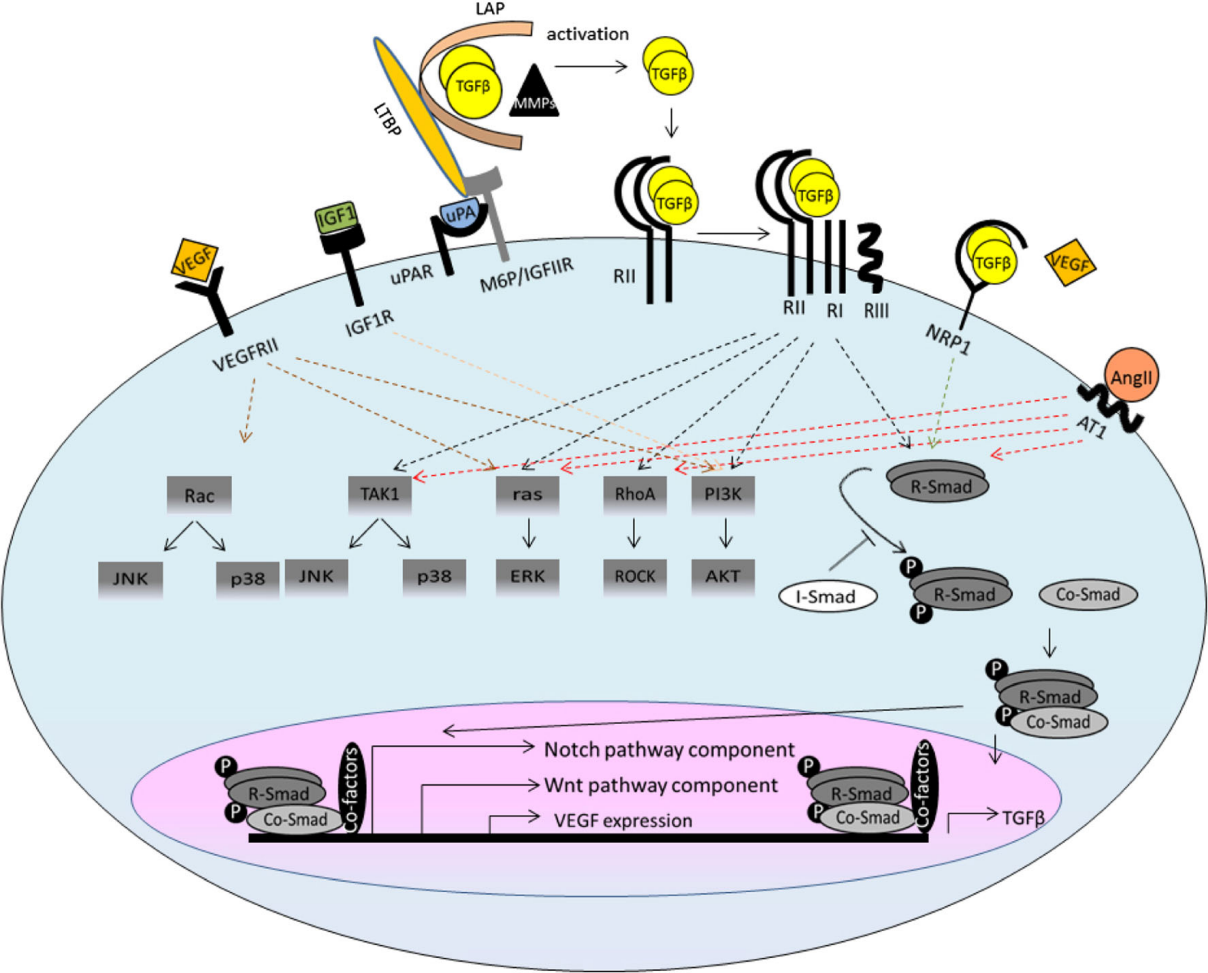
Schematic representation of TGFβ signalling and crosstalk with other signalling pathways. TGFβ ligands are synthesised as a large latent complex consisting of TGFβ dimmer covalently associated with a latency-associated peptide (LAP) and a latent TGFβ-binding protein (LTBP). The activation of latent TGFβ requires functional and physical cooperation of M6P/IGFIIR, UPAR, NRP1 and other proteases and MMPs. The released TGFβ dimers bind the type II TGFβ receptor (RII) first, which recruits and transphosphorylates the type I receptors (RI). RI propagates the signal into the cell by phosphorylating TGFβ receptor-regulated SMADs (R-Smads). They form heteromeric complexes with the common SMAD (co-Smad) and translocate to the nucleus. The R-Smads–co-Smad complex formation can be inhibited by inhibitory Smad (I-Smad). Once in the nucleus, the R-SMAD–co-SMAD complex associates with other DNA-binding transcription factors to modulate the expression of target genes. In the non-canonical pathways, the activated transforming growth factor-β (TGFβ) receptor complex transmits a signal through other factors, such as TGFβ-activated kinase 1 (TAK1), p38 mitogen-activated protein kinase (p38 MAPK), RHO, phosphoinositide 3-kinase (PI3K)–AKT, extracellular signal-regulated kinase (ERK), Rho-associated protein kinase (ROCK), or JUN N-terminal kinase (JNK). TGFβ signalling interacts extensively with other pathways, such as the WNT, Notch, AngII, IGF and VEGF pathways, which defines the context-dependent TGFβ signalling

The TGFβ family of cytokines plays important roles in embryogenesis, tissue homeostasis and regeneration (Massague 2012). Their secretion, activation and function are tightly controlled by multiple mechanisms to ensure precise signal propagation (Fig. 1). TGFβs are secreted in latent form as part of a large protein complex (Khalil 1999) and their activation requires functional and physical cooperation of mannose-6-phosphate (M6P)/insulin-like growth factor II receptor (IGFIIR), urokinase-type plasminogen activator receptor (UPAR), Neuropilin 1 (NRP1) and different proteases and metalloproteases (MMPs) (Dennis and Rifkin 1991; Scott and Firth 2004; Glinka et al. 2011; Shi et al. 2011). Once released, TGFβs bind to type II receptor TGFβRII, which recruits type I receptor, activin receptor-like kinase (ALK) (Shi and Massague 2003), and activates a multitude of intracellular signalling including canonical Smad and non-canonical ERK, JNK, TAK1, P38 and Rho cascades (Derynck and Zhang 2003). TGFβs also interact extensively with other signalling pathways leading to very different even opposite outcomes (Massague 2012).

In mammals, there are three different isoforms: TGFβ1, TGFβ2, and TGFβ3. Each of them shows distinct expression pattern and functions. TGFβ1, the focus of this review, is the predominant and most ubiquitously expressed isoform (Millan et al. 1991). In the heart, TGFβ1 regulates the signalling and function of different types of cells, including endothelial cells (ECs), vascular mural cells (pericytes in capillaries and vascular smooth muscle cells (VSMCs) in larger vessels), myofibroblasts, macrophages and cardiomyocytes (Bujak and Frangogiannis 2007; Koitabashi et al. 2011). Aberrant TGFβ1 signalling contributes to the development of a multitude of conditions associated with heart failure such as dilated and hypertrophic cardiomyopathies, post-infarction myocardial remodelling, valvular diseases and arrhythmia in both mice and humans (Cambien et al. 1996; Schultz Jel et al. 2002; Euler-Taimor and Heger 2006; Khan and Sheppard 2006; Kapur et al. 2013).

### TGFβ1 and cardiac fibrosis

TGFβ1 is a potent fibrogenic factor that mediates ECM homeostasis through different mechanisms, for example, by inducing ECM (such as collagens and fibronectin) synthesis via both canonical and non-canonical signalling cascades (Chen et al. 2000; Qiao et al. 2005; Leask 2007), decreasing the production of proteinase regulating ECM degradation (such as MMPs), promoting the production of inhibitors of these proteases (such as TIMPs) (Biernacka et al. 2011) and promoting integrin expression to increase the adhesion of cells to matrix (Thannickal et al. 2003). Studies have shown that TGFβ1 signalling pathway components, including TGFβ1, ENG and Smads, are markedly up-regulated at the site of injury after MI (Hao et al. 1999; Krum et al. 2002; Dean et al. 2005; Kapur et al. 2012), in patients suffering from hypertrophic cardiomyopathy (Villarreal and Dillmann 1992, Li et al. 1998) and dilated cardiomyopathy (Pauschinger et al. 1999; Sanderson et al. 2001), and all these conditions are characterised by excessive fibrosis in the heart. Consistently, TGFβ1 overexpression in transgenic mice leads to myocardial fibrosis (Rosenkranz et al. 2002; Seeland et al. 2002). Studies have shown that TGFβ1-mediated endothelial-to-mesenchymal transition (EndoMT) also contributes to myocardial fibrosis (Zeisberg et al. 2007; van Meeteren and ten Dijke 2012). Recently, ENG, a previously considered EC specific TGFβ1 receptor, has been found to be expressed in cardiac fibroblasts and to mediate the pro-fibrotic effect of angiotensin II (AngII) via angiotensin II receptor type 1 (AT1) (Chen et al. 2004). Reduced ENG activity led to attenuated cardiac fibrosis and increased survival in an in vivo model of heart failure (Kapur et al. 2012). This study demonstrated that the expression of ENG is significantly up-regulated in human failing left ventricles. Inhibition of the activity of ENG-attenuated TGFβ1 induced Smad2/3 phosphorylation, ECM deposition and cardiac fibrosis. *Eng* ^*+/−*^ mice with pressure overload-induced heart failure showed an increased capillary density in the heart, preserved cardiac function and improved survival. Thus, targeting the TGFβ1 signalling pathway might provide an attractive strategy to limit structural deterioration of myocardium and ultimately lead to improved cardiac function, and survival of heart failure patients.

### TGFβ1 and cardiomyocyte hypertrophy

In addition to fibrosis, hypertrophic growth of cardiomyocytes occurs in response to haemodynamic overload and represents the heart’s effort to maintain cardiac output sufficient to meet the body’s demands (Glennon et al. 1995). There is compelling evidence that TGFβ1 plays a critical role in this process. Increased TGFβ1 expression is observed in the myocardium of human patients with idiopathic hypertrophic cardiomyopathy (Li et al. 1998). Consistently, TGFβ1 overexpression in transgenic mice results in hypertrophic growth of cardiomyocytes (Rosenkranz et al. 2002). Interestingly, this TGFβ1-induced cardiac hypertrophy is associated with increased myocardial β-adrenergic receptors (ARs) density (Rosenkranz et al. 2002) and β-AR blockade treatment in TGFβ1 transgenic mice prevents cardiac hypertrophy. On the other hand, angiotensin II was shown to induce TGFβ1 expression in myocardium and TGFβ1 (Wenzel et al. 2001) is required for AngII-mediated cardiacmocyte hypertrophy (Gray et al. 1998). However, the AT1 receptor blockade is insufficient to prevent the hypertrophic response in TGFβ transgenic mice (Rosenkranz et al. 2003), and TGFβ1 knockout mice are resistant to AngII-induced cardiac hypertrophy (Schultz Jel et al. 2002). Together, these data show that TGFβ1 plays a key role in AngII-mediated growth responses of cardiomyocytes via β-AR.

### TGFβ1 and post-infarction inflammatory response

Cardiomyocyte death and hypoxia following infarction initiate inflammatory response, which leads to the infiltration of immune cells into the infarcted area, an important process for clearing debris from the wound and tissue repair (Mehta and Li 1999). In the meantime, a timely repression of inflammatory mediator synthesis is important for scar maturation. TGFβ1 plays a highly important and complex role in inflammatory response following cardiac injury (Celada and Maki 1992), It acts as a direct chemoattractant to monocytes (Wahl et al. 1987) and neutrophils (Fava et al. 1991) to recruit them to the infarct site. However, its effects on macrophages are primarily inhibitory (Frangogiannis et al. 2001). There is a need for a better understanding of the TGFβ1-modulated post-infarction inflammatory response for specific intervention that could attenuate inflammatory injury without interfering with myocardial healing.

### TGFβ1 and cardiac neovascularisation

Genetic studies in mouse and human revealed that proper TGFβ1 signalling is essential for blood vessel formation (Chang et al. 2001; Harradine and Akhurst 2006). The vascular response to TGFβ1 is highly context-dependent and is shaped by factors such as ligand bioavailability and concentration, receptor availability and internalization, cross-talk with other signalling pathways, micro/marcovessel origin of vascular cells, and cellular density (Massague 2012). Although not fully understood, it is generally considered that TGFβ1 signalling in ECs occurs through TGFβRII recruiting either the ubiquitously expressed ALK5 or the EC-specific ALK1 (possibly with ALK5). Signalling via ALK5 leads to the activation of downstream transcription factors Smad 2 and 3, and thus plays an essential role in maintaining the vasculature at quiescent state. The TGFβ1/ALK1 signalling activates Smad 1, 5 and 8, resulting in increased EC migration, proliferation and angiogenesis (Goumans et al. 2003). The two pathways interact with each other at both receptor and the Smad level (Goumans et al. 2003), with ENG as a key player in switching TGFβ signalling toward the pro-angiogenic pathway (Lebrin et al. 2004). This paper showed that ENG regulates the balance of TGFβ signalling in endothelial cells by promoting TGFβ/ALK1 and inhibiting TGFβ/ALK5 signalling, and the subsequence endothelial proliferation.

Besides ECs, blood vessels comprise another essential component. The vascular mural cells support endothelium and are involved in blood vessel maturation and homeostasis. Similar to that in ECs, TGFβ1 mediates the proliferation of VSMC in a dose-dependent manner with high-dose being inhibitory and low-dose being stimulatory (Seay et al. 2005; Tsai et al. 2009). TGFβ1 induces the contractile phenotype of VSMCs by promoting the expression of α-smooth muscle actin and smooth muscle myosin (Hautmann et al. 1997; Seay et al. 2005). Perturbed TGFβ1 signalling results in failure of VSMC recruitment (Pardali et al. 2010) and the formation of aneurysm (Choudhary et al. 2009). TGFβ1 also stimulates the expression of plasminogen activator inhibitor (PAI)-1, a potent inhibitor of matrix MMPs, and therefore promotes blood vessel maturation by preventing the degradation of provisional matrix surrounding the nascent vessel (Jain 2003). On the other hand, the juxtaposition and collaboration between mural cells and ECs are important for local activation of latent TGFβ1, which further defines its context-dependent signalling in vascular cells (Antonelliorlidge et al. 1989; Sato et al. 1990).

Together, extensive investigations have shown that TGFβ1 modulates the signalling and function of different vascular cells and participates in multiple stages of blood vessel development, which makes it an attractive target for therapeutic angiogenesis. Indeed, it has been reported that exogenous application of TGFβ1 stimulates blood vessel formation in peripheral circulation (van Royen et al. 2002). This study showed that exogenous TGFβ1 promotes peripheral collateral artery formation and collateral circulation in rabbit hind limb model of femoral artery occlusion, partly by increasing monocyte adhesion and transmigration and enhancing the expression of growth factors and cytokines. Further studies are required to evaluate the impact of TGFβ treatment on functional blood vessel formation in the heart.

### TGFβ1 signalling as a therapeutic target for heart failure?

TGFβ1 coordinates a broad spectrum of cellular processes that contributes to cardiac remodelling after MI and subsequent progression to heart failure (Bujak and Frangogiannis 2007). It can be beneficial or deleterious depending on the stage of disease development. For example, TGFβ1 plays a pivotal role in wound repair after infarction by suppressing inflammation, promoting the myofibroblast transition, and inducing blood vessel remodelling (Dobaczewski et al. 2011). However, prolonged TGFβ activation leads to excessive ECM deposition remote from the infarct site causing further damage to normal tissue architecture and cardiac function (Bujak et al. 2007). Indeed, inhibition of TGFβ before or immediately following MI led to further deterioration on cardiac function and increase mortality (Ikeuchi et al. 2004; Frantz et al. 2008), whereas its inhibition at 24 h post-MI attenuated remodelling with improved cardiac function in animal models of ischemic heart failure (Ikeuchi et al. 2004; Okada et al. 2005; Ellmers et al. 2008). In addition, due to its multifunctional and context dependent actions, a complete blockage of TGFβ signalling may cause undesirable side effects on immune regulation (Sasaki et al. 1992), angiogenesis (Bertolino et al. 2005), cancer surveillance (Salomon 2014) and wound healing (Faler et al. 2006). Taken together, TGFβ-targeted treatment must be carefully designed. A strategy that selectively attenuates the fibrogenic effect but stimulates the pro-angiogenic aspect of TGFβ1 may serve as an ideal treatment option for heart failure.

## The interaction between LRG1 and TGFβ signalling

Leucine-rich α-2 glycoprotein 1 (LRG1) is a member of leucine-rich repeat (LRR) family of proteins, many of which are involved in protein–protein interactions, signalling and cell adhesion (Ng et al. 2011). Studies have shown that differential expression of LRG1 is associated with different types of cancer (Kawakami et al. 2005; Kakisaka et al. 2007; Ferrero et al. 2009; Andersen et al. 2010; Guergova-Kuras et al. 2011; Li et al. 2011; Sandanayake et al. 2011; Ladd et al. 2012; Linden et al. 2012, 2013; Liu et al. 2012; Wu et al. 2013; He et al. 2014; Wen et al. 2014), neurodegenerative disease (Miyajima et al. 2013), inflammatory diseases (Kentsis et al. 2012; Kharbanda et al. 2012; Serada et al. 2012), hydrocephalus (Li et al. 2006, 2007; Nakajima et al. 2010, 2011), heart failure (Watson et al. 2011), autoimmune disease (Serada et al. 2010), and ageing (Nakajima et al. 2012)

We found recently that LRG1 is expressed in quiescent vasculature at low levels but is significantly up-regulated together with TGFβ1 in remodelled and neovascular vessels in the eye (Wang et al. 2013). We showed that LRG1 interacts with multiple TGFβ receptors, especially ENG, which together with TGFβ1 further promotes the ability of LRG1 to bind angiogenic ALK1 but inhibits the association between LRG1 and angiostatic ALK5. The recruitment of LRG1 into the pro-angiogenic TGFβ receptor complex leads to enhanced Smad 1, 5 phosphorylation, EC proliferation, tube formation and blood vessel outgrowth. LRG1 inhibition by genetic knockout, siRNA knockdown or neutralizing antibodies led to reduced angiogenesis. In summary, our study showed that LRG1 plays a critical role in defining the context-dependent TGFβ signalling in ECs (Wang et al. 2013).

There is evidence that LRG1 is involved in other TGFβ-regulated processes. TGFβ is known to stimulate the expression of endothelin (ET1) (Ahmedat et al. 2012), an important molecule involved in myocardial hypertrophy and fibrosis. ET1 has been shown to inhibit LRG1 expression in dermal fibroblasts suggesting a potential role of LRG1 in TGFβ-mediated fibrosis (GEO accession GDS1980 / 1417290_at / Lrg1) (Vallender and Lahn 2006).

## The involvement of LRG1 in cardiac remodelling and heart failure

### LRG1 and ageing heart

As an unavoidable process of life, different systems of the body undergo progressive structural and functional alterations, and the heart is not an exception. With ageing, there is increased plaque formation in coronary arteries, cardiac wall thickness and interstitial fibrosis (Mendes et al. 2012; Dayal et al. 2013). These structural changes of myocardium are accompanied with concurrent vascular abnormalities, such as reduced diameter and density of collateral vessels, decreased vasodilation, and increased stiffness of vessel walls (Heil and Schaper 2004; Faber et al. 2011; Wang et al. 2011). In addition, there is a decreased expression and availability of growth factors such as hypoxia-inducible factor 1α and VEGF in response to hypoxic stress (Rivard et al. 2000), and ECs become less responsive to the stimulation of angiogenic growth factors with ageing (Lahteenvuo and Rosenzweig 2012). These alterations contribute to compromised cardiac function, increased susceptibility to damage and reduced ability to repair, which subsequently lead to increased incidence of heart failure in ageing population. Two separate studies have reported an decreased expression of LRG1 in the heart of aged mice compared to that in young mice (GDS2996 / 1417290_at / Lrg1 and GDS2972 / 97420_at / Lrg1) (Reiter et al. 2007). However, it is not clear whether the reduced LRG1 expression is the cause or the consequence of ageing-related structural and functional changes in the heart. Further studies are required to explore the role of LRG1 in specific patho-physiologies of the ageing heart and to discover whether it is possible to prevent or reverse age-dependent deterioration of the heart by overexpressing LRG1.

### LRG1 and cardiac hypertrophy

Cardiac hypertrophy is the thickening of the heart wall in response to increased pressure or volume stress. Under certain conditions, such as during pregnancy or after sustained exercise, the enlargement of heart muscle is beneficial and is normally associated with a proportional increase in chamber dimensions and neovascularization (Catalucci et al. 2008). There is no concurrent fibrosis or reactivation of a foetal gene program in this physiological adaptation process (Beisvag et al. 2009). In addition, physiological hypertrophy does not cause increased risk of arrhythmia, impairment in cardiac function or future heart failure. Instead, exercise training has been shown to protect the heart against ageing-induced up-regulation of collagen deposition, collagen cross-linking, TIMP synthesis and down-regulation of active MMPs (Thomas et al. 2000, 2001; Kwak et al. 2008). The ageing-associated increase in extra-myocyte space was also significantly attenuated in rats which underwent exercise training (Kwak et al. 2008, 2011). Pathological cardiac hypertrophy, on the other hand, occurs as a consequence of hypertension, aortic stenosis, or other disease-causing stimuli. It is associated with significant structural abnormalities, which can lead to contractile dysfunction, arrhythmias and eventually heart failure (Scheuer et al. 1982; Breisch et al. 1986).

The activation of insulin-like growth factor-1 (IGF-1)/phosphoinositide 3-kinase (PI3K)/Akt pathway has been implicated in adaptive cardiac hypertrophy with endurance exercise (Neri Serneri et al. 2001). Studies have shown that transient activation of Akt1 leads to reversible cardiac hypertrophy, which is associated with reduced expression of Lrg1 (GDS2304 / 1417290_at / Lrg1) (Schiekofer et al. 2006). Consistent with this observation, a dominant negative form of PI3K significantly attenuates cardiac hypertrophy in the heart of transgenic mice overexpressing IGF1 receptor, which is also associated with a concurrent up-regulation of Lrg1 (GDS648 / 97420_at / Lrg1) (McMullen et al. 2004). However, a separate study showed no change in Lrg1 expression in transgenic mice with either constitutive active PI3K or dominant negative PI3K (GDS446 / 97420_at / Lrg1). Also, no change in Lrg1 expression was detected in the heart of rats following moderate physical training (GDS3134/1374626_at/Lrg1) (Giusti et al. 2009). These seemingly contradictory observations might be due to differences in the design of transgenic strategies, spatial and temporal expression pattern of transgenes, and the use of different animal species. Further studies are required to elucidate the role of LRG1 in physiological cardiac hypertrophy.

A reduced expression of LRG1 is observed in mouse models of pathological cardiac hypertrophy. LRG1 expression is significantly attenuated in the heart of mice with compensated pressure overload hypertrophy induced by transverse aortic constriction (TAC) (GDS794 / 97420_at / Lrg1, GDS3465 / 1417290_at / Lrg1) (Zhao et al. 2004; Smeets et al. 2008). A missense E180G mutation in α-tropomyosin (TM), an important contractile protein involved in sarcomeric function, is associated with familial hypertrophic cardiomyopathy (Chang et al. 2005). Transgenic mice overexpressing α-TM E180G exhibit severe cardiac hypertrophy characterized by myocyte disarray, asymmetric ventricular enlargement, fibrosis, cardiac arrhythmia and eventually die of heart failure (Michele et al. 2002). The expression of Lrg1 is significantly down-regulated in the ventricle of α-TM E180G transgenic mice (GDS2134 / 1417290_at / Lrg1) (Rajan et al. 2006).

Together, the literature supports lower LRG1 expression being associated with cardiac hypertrophy. Further studies are required to investigate the underlying molecular mechanism. Analysis of cardiac phenotypes in Lrg1^−/−^ with exercise or pressure overload-induced hypertrophy will provide valuable information regarding the role of LRG1 in physiological and pathological cardiac hypertrophy. LRG1 overexpression might be able to reverse cardiac hypertrophy induced by the activation of IGF1/PI3K/Akt1 pathway, exercise, pressure overload, and in α-TM E180G transgenic mice. The activation of latent TGFβ requires functional and physical cooperation of mannose-6-phosphate (M6P)/IGF II receptor (IGFIIR) and the urokinase-type plasminogen activator receptor (uPAR) (Leksa et al. 2005). Furthermore, both TGFβ1 and IGF1 signal through the PI3K/Akt pathway and there is an extensive crosstalk between the two signalling pathways during cardiac fibrosis (Butt et al. 1995), cardiomyocyte apoptosis (Hynes et al. 2009), cardiac remodelling following myocardial infarction (Stavropoulou et al. 2010) and cardiac hypertrophy (Lisa et al. 2011). Understanding the role of LRG1 in TGFβ1 and IGF1 interactions may shed new light on the molecular mechanism of cardiac hypertrophy.

### LRG1 and dilated cardiomyopathy

Muscle LIM protein (MLP) is a muscle-restricted cytoskeletal binding protein. The down-regulation of MLP protein is observed in human patients with idiopathic-dilated cardiomyopathy (Zolk et al. 2000). Consistent with this observation, MLP^−/−^ mice develop dilated cardiomyopathy and eventually heart failure (Arber et al. 1997). Calsequestrin (CSQ) is a high-capacity sarcoplasmic reticulum Ca^2+^ binding protein. The myocardial-targeted overexpression of CSQ also leads to heart failure associated with dilated cardiomyopathy and left ventricular dysfunction (Jones et al. 1998). Both mouse models exhibited many key features present in the failing heart in human, such as functional β-AR uncoupling (Rockman et al. 1998). Advanced heart failure is normally developed in 6-month old MLP^−/−^ mice and 14-week old CSQ transgenic mice. Despite different aetiologies, a decreased Lrg1 expression is associated with deterioration of cardiac function with lowest Lrg1 expression observed at the advanced stage of heart failure in both mouse models (GDS411 / aa172851_s_at / Lrg1) (Blaxall et al. 2003). However, the molecular basis of LRG1 down-regulation in both mouse models remains to be resolved, which is vital for dissecting the mechanism of dilated cardiomyopathy pathogenesis. LRG1 overexpression in myocardium might restore cardiac function and slow down the progression of heart failure in both models. Information extracted from this study will assist the designing effective treatment for dilated cardiomyopathy associated heart failure.

### LRG1 and hypertension

Hypertension is a major risk factor for heart failure. To work against the high pressure, the heart must pump harder, which may lead to left ventricular hypertrophy and heart failure over time. The S.LWEx12x2x3x5 congenic rat is a model for hypertension and exhibits concentric cardiac hypertrophy. The expression of LRG1 is significantly down-regulated in the left ventricle of hypertensive S.LWEx12x2x3x5 rat exhibiting concentric cardiac hypertrophy with augmented contractile function (GDS3873 _ 1374626_at _ Lrg1) (Gopalakrishnan et al. 2011). However, it is not clear whether hypertension has a direct impact on the expression of Lrg1 or mediates LRG1 expression indirectly via hypertension-induced compensation. To study the expression of Lrg1 in other hypertension animal models with or without myocardial abnormalities will enrich our understanding of direct association between LRG1 and hypertension aetiology and development. LRG1 overexpression at the right dosage and right timing might prevent or reverse hypertension and hypertension-induced cardiomyopathy.

## Perspectives

There is compelling evidence to suggest that TGFβ1 is associated with various cardiac pathologies involved in heart failure. Targeting TGFβ1 therefore represents an attractive strategy in managing progression of the disease. A number of therapeutic approaches for blocking the actions of TGFβ1 have been suggested, such as TGFβ1 neutralizing antibody (Kuwahara et al. 2002), soluble TGFβ receptor II (Okada et al. 2005), and small molecule inhibitors (Engebretsen et al. 2014). Some of these successfully attenuated cardiac fibrosis, decreased ventricular chamber dilation, improved cardiac function, and reduced mortality after infarction in preclinical studies (Kuwahara et al. 2002; Ellmers et al. 2008; Lian et al. 2010). However, given its role in angiogenesis, targeting TGFβ may affect collateral and microvessel formation, remodelling and perfusion following MI and cause increased burden of ischemic tissue. In addition, TGFβ1 is a pleiotropic cytokine with vital homeostatic functions. Broad TGFβ inhibition is likely to have adverse side effects, such as the development of autoimmune diseases, delayed wound healing, and tumour formation. Selectively targeting specific disease-driving aspects of TGFβ signalling at the right dose and timing and for an appropriate period is therefore critical in producing desirable therapeutic effects.

**Fig. 2:**
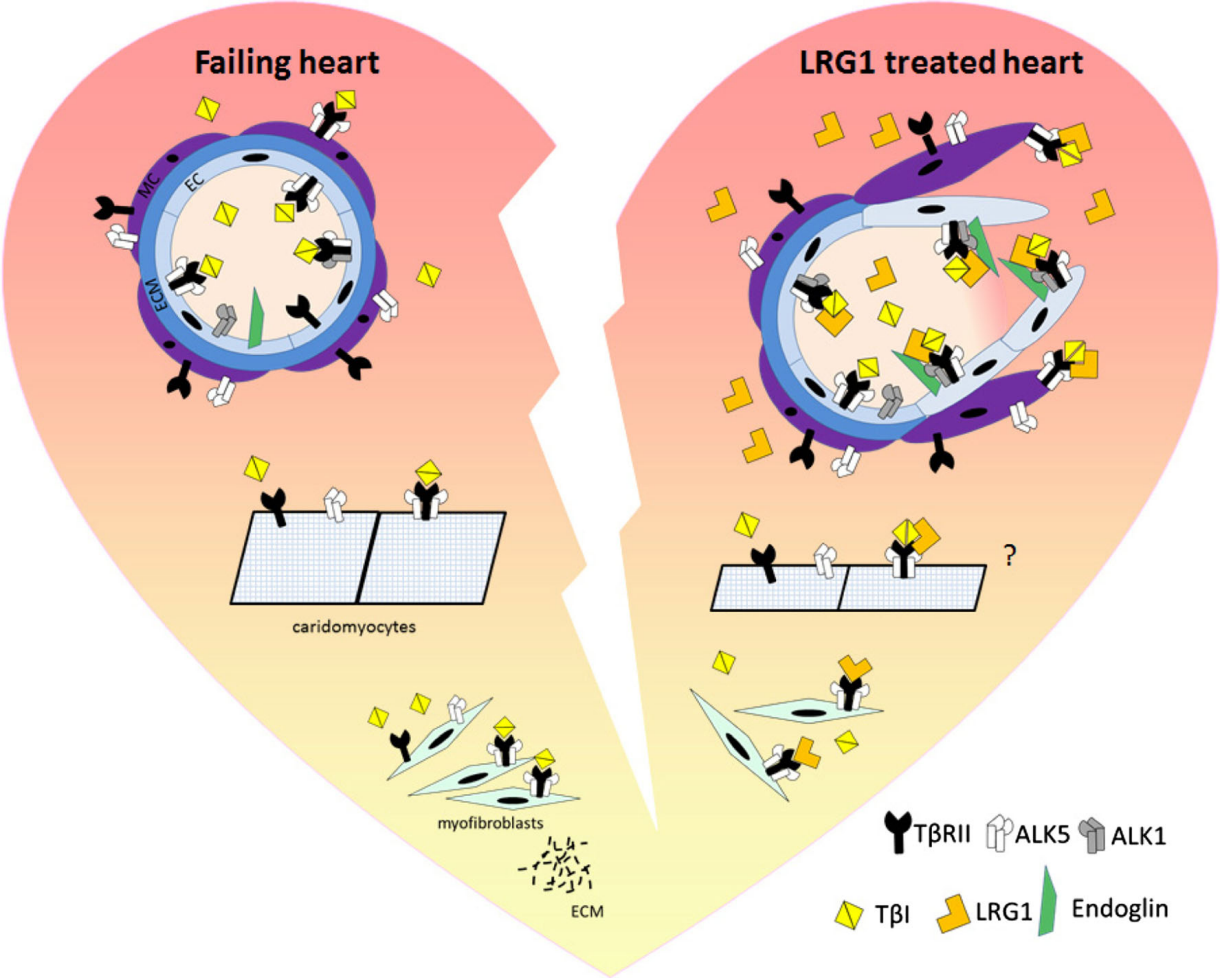
Potential role of LRG1 in cardiac remodelling. In the failing heart, the ability of blood vessels to respond to angiogenic factors is compromised, fibroblasts acquire myofibroblast phenotype by expressing increased ECM protein, and cardiomycytes are enlarged and undergo increased apoptosis. TGFβ1 mainly signals through ALK5 in ECs, cardiomyocyte and myofibroblasts leading to cardiac remodelling. In LRG1-treated heart, LRG1 switches TGFβ1 signalling towards the proangiogenic ALK1 signalling in ECs and promotes blood vessel formation. In myofibroblasts, LRG1 competes with TGFβ to bind ALK5 and antagonise TGFβ-induced ECM synthesis and to prevent fibrosis. With the presence of LRG1, there is reduced cardiomyocyte apoptosis and size

Our recent study led to the identification of a novel angiogenic factor, LRG1. In addition to ECs, LRG1 is also expressed in cardiac fibroblasts (Ifkovits et al. 2014) and cardiomyocytes (Chen et al. 2004). It is likely that LRG1 mediates TGFβ signalling through the ubiquitously expressed type I TGFβ receptor, ALK5, in non-ECs. There is convincing evidence that a decreased expression of LRG1 is associated with increased fibrosis, aberrant vascular properties, and altered cardiomyocytes characteristics in ageing heart, and in failing heart induced by genetic modification, pressure overload, or hypertension (Fig. 2). Understanding the molecular mechanism underlying the role of LRG1 and its regulation in cardiac remodelling process will assist the development of novel treatments for heart failure.

### LRG1, a potential target for therapeutic angiogenesis

LRG1 promotes EC proliferation, tube formation and vessel outgrowth through regulating the endothelial TGFβ signalling (Wang et al. 2013). As it binds to the ubiquitously expressed type I TGFβ receptor, ALK5, LRG1 might mediate the signalling and function of other types of vascular cells and regulate blood vessel remodelling. TGFβ1 interacts with VEGF signalling at the receptor level (Glinka et al. 2011) and regulates VEGF expression in macrophages (Jeon et al. 2007) and ECs (Ferrari et al. 2006). Understanding the role of LRG1 in TGFβ1 and VEGF crosstalk may provide valuable information regarding the molecular mechanism of angiogenesis, and facilitate the development of novel therapeutic angiogenesis strategies. Angiogenic factors are normally released in a tightly controlled and timely manner. Lessons learned from previous studies suggested that the hypoxia condition of the ischemic tissue during treatment dramatically affects the benefit of therapeutic angiogenesis. Virus-based transgene delivery systems offer an opportunity for site-specific administration at the right location, time and dose. The route of delivery may also affect the efficacy of treatment. Direct delivery to the myocardium may be an effective method for therapeutic angiogenesis in the heart. Comorbidities are known to have a great impact on the outcome of therapeutic angiogenesis. It is therefore important to evaluate the impact of LRG1 on angiogenesis under heart failure-associated disease conditions such as diabetes, hypertension and obesity. As oedema can impose further burdens on ischemic tissue, studies are required to investigate the impact of LRG1 on blood permeability. It is worth noting that excessive angiogenesis contributes to cancer growth and metastasis, atherosclerotic plaque expansion and instability, arthritis and blinding eye diseases. Understanding the involvement of LRG1 in other vascular complications will help to predict potential side effects of the treatment. Taken together, LRG1 is an attractive target for therapeutic angiogenesis. Further studies are required to evaluate the efficacy and toxicity of LRG1 treatment for heart failure.

### LRG1, a potential modulator of cardiac remodelling process

Cardiac fibrosis is a key contributor to the morbidity and mortality in heart failure. Despite intense research efforts, no effective treatment is available to control this detrimental process. Evidence showed that a reduced expression of LRG1 is associated with an increased TGFβ1 activity during the cardiac remodelling process in response to injury. However, it is not clear whether TGFβ1 exerts its function by inhibiting the expression of LRG1. As LRG1 binds ALK5 and TGFβRII independently of TGFβ1, it may exert its function by competing with TGFβ1 for binding with the ALK5/TGFβRII receptor complex. In addition to its role in mediating TGFβ1 signalling in endothelial cells, ENG plays an important role in cardiac fibrosis (Kapur et al. 2012). Unveiling the interaction between ENG and LRG1 in fibrosis will advance our understanding of the molecular mechanism underlying the cardiac remodelling process. It is widely accepted that context-dependent TGFβ1 signalling is defined by extensive interaction with other signalling pathways including IGF1 and AngII. Understanding the involvement of LRG1 in this highly complex network will shed light on the molecular mechanism of cardiac remodelling. Studies have shown that non-ECs including cardiac fibroblasts and cardiomyocytes also express LRG1. It will be interesting to see novel binding partners of LRG1 in these cells, and it will help to elucidate the TGFβ1-independent role of LRG1 in non-ECs in the heart. On the other hand, ECM is important for maintaining atherosclerotic plaque stability. Augment of collagen degradation has been correlated with ruptured plaques in patients (Cheng et al. 2009). Further studies are required to unveil the molecular and cellular mechanism of LRG1 in atherosclerotic plaque development and progression, which will provide valuable insights into the potential side effects of LRG1-targeted treatment.

## Summary

At the moment, the focus of heart failure treatment is directed at risk factor management and alleviating symptoms; however, not all patients are suitable or will benefit from current therapeutics. As the magnitude of heart failure continues to accelerate globally, there is a pressing need for new treatments. Direct intervention on structural abnormalities of myocardium is a promising strategy. A reduced expression of LRG1 is associated with cardiac remodelling characterised by hypertrophy, fibrosis, and abnormal vasculature in various conditions leading to heart failure. However, it is not clear whether altered LRG1 expression is the cause or the consequence of these detrimental processes, or how LRG1 is correlated with specific pathologies and evolution of the disease. Although a great deal of work is still needed to fully understand the underlying mechanisms, targeting LRG1 and its regulators might offer a unique approach to treating heart failure by simultaneously targeting different pathologies of the disease.

## References

[CR1] Ahmedat AS (2012). beta(2)-adrenoceptors and muscarinic receptors mediate opposing effects on endothelin-1 expression in human lung fibroblasts. Eur J Pharmacol.

[CR2] Andersen JD, et al. (2010) Leucine-rich alpha-2-glycoprotein-1 is upregulated in sera and tumors of ovarian cancer patients. J Ovarian Res 3: 21 10.1186/1757-2215-3-21 PMC2949730 20831812

[CR3] Antonelliorlidge A (1989). An activated form of transforming growth factor-beta is produced by cocultures of endothelial-cells and pericytes. Proc Natl Acad Sci U S A.

[CR4] Antoniucci D (2002). Relation of time to treatment and mortality in patients with acute myocardial infarction undergoing primary coronary angioplasty. Am J Cardiol.

[CR5] Arber S (1997). MLP-deficient mice exhibit a disruption of cardiac cytoarchitectural organization, dilated cardiomyopathy, and heart failure. Cell.

[CR6] Banerjee I (2006). Dynamic interactions between myocytes, fibroblasts, and extracellular matrix. Ann N Y Acad Sci.

[CR7] Beisvag V (2009). Pathological and physiological hypertrophies are regulated by distinct gene programs. Eur J Cardiovasc Prev Rehabil.

[CR8] Bertolino P (2005). Transforming growth factor-beta signal transduction in angiogenesis and vascular disorders. Chest.

[CR9] Biernacka A (2011). TGF-beta signaling in fibrosis. Growth Factors.

[CR10] Blaxall BC (2003). Differential myocardial gene expression in the development and rescue of murine heart failure. Physiol Genomics.

[CR11] Breisch EA (1986). Exercise-induced cardiac hypertrophy: a correlation of blood flow and microvasculature. J Appl Physiol (1985).

[CR12] Bujak M, Frangogiannis NG (2007). The role of TGF-beta signaling in myocardial infarction and cardiac remodeling. Cardiovasc Res.

[CR13] Bujak M (2007). Essential role of Smad3 in infarct healing and in the pathogenesis of cardiac remodeling. Circulation.

[CR14] Butt RP (1995). Collagen production and replication by cardiac fibroblasts is enhanced in response to diverse classes of growth-factors. Eur J Cell Biol.

[CR15] Cambien F (1996). Polymorphisms of the transforming growth factor-beta 1 gene in relation to myocardial infarction and blood pressure. The Etude Cas-Temoin de l’Infarctus du Myocarde (ECTIM) Study. Hypertension.

[CR16] Cao Y (2009). Monotherapy versus combination therapy of angiogenic and arteriogenic factors for the treatment of ischemic disorders. Curr Mol Med.

[CR17] Cao Y (2005). Update on therapeutic neovascularization. Cardiovasc Res.

[CR18] Cao R (2010). VEGFR1-mediated pericyte ablation links VEGF and PlGF to cancer-associated retinopathy. Proc Natl Acad Sci U S A.

[CR19] Carmeliet P, Jain RK (2011). Molecular mechanisms and clinical applications of angiogenesis. Nature.

[CR20] Catalucci D (2008). Physiological myocardial hypertrophy: how and why?. Front Biosci.

[CR21] Celada A, Maki RA (1992). Transforming growth factor-beta enhances the M-CSF and GM-CSF-stimulated proliferation of macrophages. J Immunol.

[CR22] Chang H (2001). Studying TGF-beta superfamily signaling by knockouts and knockins. Mol Cell Endocrinol.

[CR23] Chang AN (2005). Functional consequences of hypertrophic and dilated cardiomyopathy-causing mutations in alpha-tropomyosin. J Biol Chem.

[CR24] Chen MM (2000). CTGF expression is induced by TGF- beta in cardiac fibroblasts and cardiac myocytes: a potential role in heart fibrosis. J Mol Cell Cardiol.

[CR25] Chen H (2004). Gene expression changes associated with fibronectin-induced cardiac myocyte hypertrophy. Physiol Genomics.

[CR26] Cheng C (2009). Activation of MMP8 and MMP13 by angiotensin II correlates to severe intra-plaque hemorrhages and collagen breakdown in atherosclerotic lesions with a vulnerable phenotype. Atherosclerosis.

[CR27] Choi JH (2013). Frequency of myocardial infarction and its relationship to angiographic collateral flow in territories supplied by chronically occluded coronary arteries. Circulation.

[CR28] Choudhary B (2009). Absence of TGFbeta signaling in embryonic vascular smooth muscle leads to reduced lysyl oxidase expression, impaired elastogenesis, and aneurysm. Genesis.

[CR29] Cohn JN (2000). Cardiac remodeling–concepts and clinical implications: a consensus paper from an international forum on cardiac remodeling. Behalf of an International Forum on Cardiac Remodeling. J Am Coll Cardiol.

[CR30] Cuevas P (1991). Hypotensive activity of fibroblast growth factor. Science.

[CR31] Dayal S (2013). Hydrogen peroxide promotes ageing-related platelet hyperactivation and thrombosis.. Circulation.

[CR32] Dean RG (2005). Connective tissue growth factor and cardiac fibrosis after myocardial infarction. J Histochem Cytochem.

[CR33] Dennis PA, Rifkin DB (1991). Cellular activation of latent transforming growth factor beta requires binding to the cation-independent mannose 6-phosphate/insulin-like growth factor type II receptor. Proc Natl Acad Sci U S A.

[CR34] Derynck R, Zhang YE (2003). Smad-dependent and Smad-independent pathways in TGF-beta family signalling. Nature.

[CR35] Dobaczewski M (2011). Transforming growth factor (TGF)-beta signaling in cardiac remodeling. J Mol Cell Cardiol.

[CR36] Ellmers LJ (2008). Transforming growth factor-beta blockade down-regulates the renin-angiotensin system and modifies cardiac remodeling after myocardial infarction. Endocrinology.

[CR37] Engebretsen KV (2014). Attenuated development of cardiac fibrosis in left ventricular pressure overload by SM16, an orally active inhibitor of ALK5. J Mol Cell Cardiol.

[CR38] Euler-Taimor G, Heger J (2006). The complex pattern of SMAD signaling in the cardiovascular system. Cardiovasc Res.

[CR39] Faber JE (2011). Ageing causes collateral rarefaction and increased severity of ischemic injury in multiple tissues. Arterioscler Thromb Vasc Biol.

[CR40] Faler BJ (2006). Transforming growth factor-beta and wound healing. Perspect Vasc Surg Endovasc Ther.

[CR41] Fava RA (1991). Transforming growth factor beta 1 (TGF-beta 1) induced neutrophil recruitment to synovial tissues: implications for TGF-beta-driven synovial inflammation and hyperplasia. J Exp Med.

[CR42] Ferrari G (2006). VEGF, a prosurvival factor, acts in concert with TGF-beta 1 to induce endothelial cell apoptosis. Proc Natl Acad Sci U S A.

[CR43] Ferrero S (2009). Increased expression of one isoform of leucine-rich alpha-2-glycoprotein in peritoneal fluid of women with uterine leiomyomas. Arch Gynecol Obstet.

[CR44] Forouzanfar MH (2013). Prevalence of heart failure by cause in 21 regions: global burden of diseases, injuries and risk factors-2010 study. J Am Coll Cardiol.

[CR45] Fortuin FD (2003). One-year follow-up of direct myocardial gene transfer of vascular endothelial growth factor-2 using naked plasmid deoxyribonucleic acid by way of thoracotomy in no-option patients. Am J Cardiol.

[CR46] Frangogiannis NG (2001). Induction and suppression of interferon-inducible protein (IP)-10 in reperfused myocardial infarcts may regulate angiogenesis.. Faseb J.

[CR47] Frantz S (2008). Transforming growth factor beta inhibition increases mortality and left ventricular dilatation after myocardial infarction. Basic Res Cardiol.

[CR48] Giusti B (2009). Gene expression profile of rat left ventricles reveals persisting changes following chronic mild exercise protocol: implications for cardioprotection. BMC Genomics.

[CR49] Glennon PE (1995). Cellular mechanisms of cardiac hypertrophy. Br Heart J.

[CR50] Glinka Y (2011). Neuropilin-1 exerts co-receptor function for TGF-beta-1 on the membrane of cancer cells and enhances responses to both latent and active TGF-beta. Carcinogenesis.

[CR51] Gopalakrishnan K (2011). Augmented rififylin is a risk factor linked to aberrant cardiomyocyte function, short-QT interval and hypertension.. Hypertension.

[CR52] Goumans MJ (2003). Controlling the angiogenic switch: a balance between two distinct TGF-b receptor signaling pathways. Trends Cardiovasc Med.

[CR53] Gray MO (1998). Angiotensin II stimulates cardiac myocyte hypertrophy via paracrine release of TGF-beta 1 and endothelin-1 from fibroblasts. Cardiovasc Res.

[CR54] Grines CL (2002). Angiogenic Gene Therapy (AGENT) trial in patients with stable angina pectoris. Circulation.

[CR55] Grines CL (2003). A randomized, double-blind, placebo-controlled trial of Ad5FGF-4 gene therapy and its effect on myocardial perfusion in patients with stable angina. J Am Coll Cardiol.

[CR56] Guergova-Kuras M (2011). Discovery of lung cancer biomarkers by profiling the plasma proteome with monoclonal antibody libraries.. Mol Cell Proteomics.

[CR57] Habib GB (1991). Influence of coronary collateral vessels on myocardial infarct size in humans. Results of phase I thrombolysis in myocardial infarction (TIMI) trial. The TIMI Investigators. Circulation.

[CR58] Hao J (1999). Elevation of expression of Smads 2, 3, and 4, decorin and TGF-beta in the chronic phase of myocardial infarct scar healing. J Mol Cell Cardiol.

[CR59] Harada K (1994). Basic fibroblast growth factor improves myocardial function in chronically ischemic porcine hearts. J Clin Invest.

[CR60] Hariawala MD (1996). VEGF improves myocardial blood flow but produces EDRF-mediated hypotension in porcine hearts. J Surg Res.

[CR61] Harradine KA, Akhurst RJ (2006). Mutations of TGF beta signaling molecules in human disease. Ann Med.

[CR62] Hautmann MB (1997). A transforming growth factor beta (TGFbeta) control element drives TGFbeta-induced stimulation of smooth muscle alpha-actin gene expression in concert with two CArG elements. J Biol Chem.

[CR63] He X (2014). Screening differential expression of serum proteins in AFP-negative HBV-related hepatocellular carcinoma using iTRAQ -MALDI-MS/MS. Neoplasma.

[CR64] Hedlund EM (2009). Malignant cell-derived PlGF promotes normalization and remodeling of the tumor vasculature. Proc Natl Acad Sci U S A.

[CR65] Heil M, Schaper W (2004). Influence of mechanical, cellular, and molecular factors on collateral artery growth (arteriogenesis). Circ Res.

[CR66] Heinl-Green A (2005). The efficacy of a ‘master switch gene’ HIF-1 alpha in a porcine model of chronic myocardial ischaemia. Eur Heart J.

[CR67] Hendel RC (2000). Effect of intracoronary recombinant human vascular endothelial growth factor on myocardial perfusion: evidence for a dose-dependent effect. Circulation.

[CR68] Henry TD (2001). Intracoronary administration of recombinant human vascular endothelial growth factor to patients with coronary artery disease. Am Heart J.

[CR69] Heuser R (2000). A retrospective study of 6,671 patients comparing coronary stenting and balloon angioplasty. J Invasive Cardiol.

[CR70] Ho CY (2010). Myocardial fibrosis as an early manifestation of hypertrophic cardiomyopathy. N Engl J Med.

[CR71] Horowitz JR (1997). Vascular endothelial growth factor/vascular permeability factor produces nitric oxide-dependent hypotension. Evidence for a maintenance role in quiescent adult endothelium. Arterioscler Thromb Vasc Biol.

[CR72] Horvath KA (2000). Transmyocardial laser revascularization in the treatment of myocardial ischemia. J Card Surg.

[CR73] Hynes B (2009). Endothelial progenitor cell derived conditioned media reduces in vivo cardiomyocyte apoptosis acting through TGFB1 and IGF1. J Am Coll Cardiol.

[CR74] Ifkovits JL (2014). Inhibition of TGFbeta signaling increases direct conversion of fibroblasts to induced cardiomyocytes. PLoS ONE.

[CR75] Ikeuchi M (2004). Inhibition of TGF-beta signaling exacerbates early cardiac dysfunction but prevents late remodeling after infarction. Cardiovasc Res.

[CR76] Inoue M (1998). Vascular endothelial growth factor (VEGF) expression in human coronary atherosclerotic lesions: possible pathophysiological significance of VEGF in progression of atherosclerosis. Circulation.

[CR77] Iredale JP (2007). Models of liver fibrosis: exploring the dynamic nature of inflammation and repair in a solid organ. J Clin Invest.

[CR78] Jain RK (2003). Molecular regulation of vessel maturation. Nat Med.

[CR79] Jeon SH (2007). Mechanisms underlying TGF-beta 1-induced expression of VEGF and Flk-1 in mouse macrophages and their implications for angiogenesis. J Leukoc Biol.

[CR80] Johnson FL (2014). Pathophysiology and etiology of heart failure. Cardiol Clin.

[CR81] Jones LR (1998). Regulation of Ca2+ signaling in transgenic mouse cardiac myocytes overexpressing calsequestrin. J Clin Invest.

[CR82] Kakisaka T (2007). Plasma proteomics of pancreatic cancer patients by multi-dimensional liquid chromatography and two-dimensional difference gel electrophoresis (2D-DIGE): up-regulation of leucine-rich alpha-2-glycoprotein in pancreatic cancer. J Chromatogr B.

[CR83] Kapur NK (2012). Reduced endoglin activity limits cardiac fibrosis and improves survival in heart failure. Circulation.

[CR84] Kapur NK (2013). Endoglin: a critical mediator of cardiovascular health. Vasc Health Risk Manag.

[CR85] Karagueuzian HS (2011). Targeting cardiac fibrosis: a new frontier in antiarrhythmic therapy?. Am J Cardiovasc Dis.

[CR86] Kastrup J (2011). A randomised, double-blind, placebo-controlled, multicentre study of the safety and efficacy of BIOBYPASS (AdGVVEGF121.10NH) gene therapy in patients with refractory advanced coronary artery disease: the NOVA trial. EuroIntervention.

[CR87] Kaul U (1991). Restenosis after successful coronary angioplasty in single vessel disease. Indian Heart J.

[CR88] Kawakami T (2005). Proteomic analysis of sera from hepatocellular carcinoma patients after radiofrequency ablation treatment. Proteomics.

[CR89] Kehat I, Molkentin JD (2010). Molecular pathways underlying cardiac remodeling during pathophysiological stimulation. Circulation.

[CR90] Kentsis A (2012). Detection and diagnostic value of urine leucine-rich alpha-2-glycoprotein in children with suspected acute appendicitis. Ann Emerg Med.

[CR91] Khalil N (1999) TGF-beta: from latent to active. Microbes Infect 1:1255–1263 10.1016/s1286-4579(99)00259-2 10611753

[CR92] Khan R, Sheppard R (2006). Fibrosis in heart disease: understanding the role of transforming growth factor-beta in cardiomyopathy, valvular disease and arrhythmia. Immunology.

[CR93] Kharbanda AB (2012). Novel serum and urine markers for pediatric appendicitis. Acad Emerg Med.

[CR94] Kim I (2001). Vascular endothelial growth factor expression of intercellular adhesion molecule 1 (ICAM-1), vascular cell adhesion molecule 1 (VCAM-1), and E-selectin through nuclear factor-kappa B activation in endothelial cells. J Biol Chem.

[CR95] Koitabashi N (2011). Pivotal role of cardiomyocyte TGF-beta signaling in the murine pathological response to sustained pressure overload. J Clin Invest.

[CR96] Krum H (2002). Which factors mediate cardiac fibrosis following myocardial infarction? Role of TGF beta 1 & CTGF in pathological collagen deposition post-MI. Circulation.

[CR97] Kuwahara F (2002). Transforming growth factor-beta function blocking prevents myocardial fibrosis and diastolic dysfunction in pressure-overloaded rats.. Circulation.

[CR98] Kwak HB (2008). Exercise training attenuates extracellular matrix remodeling in the ageing Rat heart. Med Sci Sports Exerc.

[CR99] Kwak HB (2011). Exercise training reduces fibrosis and matrix metalloproteinase dysregulation in the ageing rat heart. Faseb J.

[CR100] Ladd JJ (2012). Increased plasma levels of the APC-interacting protein MAPRE1, LRG1, and IGFBP2 preceding a diagnosis of colorectal cancer in women. Cancer Prev Res (Phila).

[CR101] Lahteenvuo J, Rosenzweig A (2012). Effects of ageing on angiogenesis. Circ Res.

[CR102] Landau C (1995). Intrapericardial basic fibroblast growth-factor induces myocardial angiogenesis in a rabbit model of chronic ischemia. Am Heart J.

[CR103] Lazarous DF (1996). Comparative effects of basic fibroblast growth factor and vascular endothelial growth factor on coronary collateral development and the arterial response to injury. Circulation.

[CR104] Leask A (2007). TGFbeta, cardiac fibroblasts, and the fibrotic response. Cardiovasc Res.

[CR105] Lebrin F (2004). Endoglin promotes endothelial cell proliferation and TGF-beta/ALK1 signal transduction. EMBO J.

[CR106] Lee LY (2000). Focal angiogen therapy using intramyocardial delivery of an adenovirus vector coding for vascular endothelial growth factor 121. Ann Thorac Surg.

[CR107] Leksa V (2005). TGF-beta-induced apoptosis in endothelial cells mediated by M6P/IGFII-R and mini-plasminogen. J Cell Sci.

[CR108] Li G (1998). Elevated insulin-like growth factor-I and transforming growth factor-beta 1 and their receptors in patients with idiopathic hypertrophic obstructive cardiomyopathy. A possible mechanism.. Circulation.

[CR109] Li X (2006). Analysis of potential diagnostic biomarkers in cerebrospinal fluid of idiopathic normal pressure hydrocephalus by proteomics. Acta Neurochir (Wien).

[CR110] Li X (2007). Expression of TGF-betas and TGF-beta type II receptor in cerebrospinal fluid of patients with idiopathic normal pressure hydrocephalus. Neurosci Lett.

[CR111] Li Y (2011). Proteomic identification of exosomal LRG1: a potential urinary biomarker for detecting NSCLC. Electrophoresis.

[CR112] Lian RQ (2010). Soluble transforming growth factor-beta 1 receptor II might inhibit transforming growth factor-beta-induced myofibroblast differentiation and improve ischemic cardiac function after myocardial infarction in rats. Coron Artery Dis.

[CR113] Linden M (2012). Proteomic analysis of urinary biomarker candidates for nonmuscle invasive bladder cancer. Proteomics.

[CR114] Linden M (2013). Tumour expression of bladder cancer-associated urinary proteins. BJU Int.

[CR115] Lisa M (2011). Insulin-Like Growth Factor-1 (IGF-1) Reduces ischemic changes and increases circulating angiogenic factors in experimentally - induced myocardial infarction in rats. Vasc Cell.

[CR116] Liu YS (2012). Shotgun and targeted proteomics reveal that pre-surgery serum levels of LRG1, SAA, and C4BP may refine prognosis of resected squamous cell lung cancer. J Mol Cell Biol.

[CR117] Lopez JJ (1998). Angiogenic potential of perivascularly delivered aFGF in a porcine model of chronic myocardial ischemia. Am J Physiol.

[CR118] Lopez B (2001). Biochemical assessment of myocardial fibrosis in hypertensive heart disease. Hypertension.

[CR119] Losordo DW (1998). Gene therapy for myocardial angiogenesis - Initial clinical results with direct myocardial injection of phVEGF(165) as sole therapy for myocardial ischemia. Circulation.

[CR120] Losordo DW (2002). Phase 1/2 placebo-controlled, double-blind, dose-escalating trial of myocardial vascular endothelial growth factor 2 gene transfer by catheter delivery in patients with chronic myocardial ischemia. Circulation.

[CR121] Manabe I (2002). Gene expression in fibroblasts and fibrosis: involvement in cardiac hypertrophy. Circ Res.

[CR122] Mann DL, Chakinala M (2012) Heart failure and Cor Pulmonale. Chapter 234 In: Harrison's Internal Medicine. McGraw-Hill, New York

[CR123] Massague J (2012). TGFbeta signalling in context. Nat Rev Mol Cell Biol.

[CR124] McMullen JR (2004). Deletion of ribosomal S6 kinases does not attenuate pathological, physiological, or insulin-like growth factor 1 receptor-phosphoinositide 3-kinase-induced cardiac hypertrophy. Mol Cell Biol.

[CR125] Mehta JL, Li DY (1999). Inflammation in ischemic heart disease: response to tissue injury or a pathogenetic villain?. Cardiovasc Res.

[CR126] Meier P (2012). The impact of the coronary collateral circulation on mortality: a meta-analysis. Eur Heart J.

[CR127] Mendes AB (2012). Quantification of left ventricular myocardial collagen system in children, young adults, and the elderly. Med (B Aires).

[CR128] Michele DE (2002). Cardiac dysfunction in hypertrophic cardiomyopathy mutant tropomyosin mice is transgene-dependent, hypertrophy-independent, and improved by beta-blockade. Circ Res.

[CR129] Millan FA (1991). Embryonic gene expression patterns of TGF beta 1, beta 2 and beta 3 suggest different developmental functions in vivo. Development.

[CR130] Miyajima M et al. (2013). Leucine-Rich alpha 2-Glycoprotein Is a Novel Biomarker of Neurodegenerative Disease in Human Cerebrospinal Fluid and Causes Neurodegeneration in Mouse Cerebral Cortex. PLoS ONE 8(9):e74453 10.1371/journal.pone.0074453 PMC3776841 24058569

[CR131] Nagy JA (2007). VEGF-A and the induction of pathological angiogenesis. Annu Rev Pathol.

[CR132] Nakajima M (2010). Diagnostic value of CSF biomarker profile in idiopathic normal pressure hydrocephalus; leucine-rich alpha-2-glycoprotein is a potential biological marker. Rinsho Shinkeigaku.

[CR133] Nakajima M (2011). Leucine-rich alpha-2-glycoprotein is a marker for idiopathic normal pressure hydrocephalus. Acta Neurochir.

[CR134] Nakajima M (2012). Brain localization of leucine-rich alpha 2-glycoprotein and its role. Acta Neurochir Suppl.

[CR135] Neri Serneri G, Boddi M, Modesti P, Cecioni I, Coppo M, Padeletti L, Michelucci A, Colella A, Galanti G (2001) Increased cardiac sympathetic activity and insulin-like growth factor-I formation are associated with physiological hypertrophy in athletes. Circ Res 89:977–982 10.1161/hh2301.100982 11717153

[CR136] Ng AC (2011). Human leucine-rich repeat proteins: a genome-wide bioinformatic categorization and functional analysis in innate immunity. Proc Natl Acad Sci U S A.

[CR137] Nishimura RA (2014). 2014 AHA/ACC guideline for the management of patients with valvular heart disease: a report of the american college of cardiology/american heart association task force on practice guidelines. Circulation.

[CR138] Norgren L (2007). Inter-society consensus for the management of peripheral arterial disease (TASC II).. J Vasc Surg.

[CR139] Okada H (2005). Postinfarction gene therapy against transforming growth factor-beta signal modulates infarct tissue dynamics and attenuates left ventricular remodeling and heart failure. Circulation.

[CR140] Pardali E (2010). Signaling by members of the TGF-beta family in vascular morphogenesis and disease. Trends Cell Biol.

[CR141] Pauschinger M (1999). Dilated cardiomyopathy is associated with significant changes in collagen type I/III ratio. Circulation.

[CR142] Qiao B (2005). Transforming growth factor (TGF)-beta-activated kinase 1 mimics and mediates TGF-beta-induced stimulation of type II collagen synthesis in chondrocytes independent of Col2a1 transcription and Smad3 signaling. J Biol Chem.

[CR143] Rajan S (2006). Microarray analysis of gene expression during early stages of mild and severe cardiac hypertrophy. Physiol Genomics.

[CR144] Reilly JP (2005). Long-term (2-year) clinical events following transthoracic intramyocardial gene transfer of VEGF-2 in no-option patients. J Interv Cardiol.

[CR145] Reiter E (2007). Anti-inflammatory properties of alpha- and gamma-tocopherol. Mol Asp Med.

[CR146] Rivard A (2000). Age-dependent defect in vascular endothelial growth factor expression is associated with reduced hypoxia-inducible factor 1 activity. J Biol Chem.

[CR147] Rockman HA (1998). Expression of a beta-adrenergic receptor kinase 1 inhibitor prevents the development of myocardial failure in gene-targeted mice. Proc Natl Acad Sci U S A.

[CR148] Rosengart TK (1999). Angiogenesis gene therapy: phase I assessment of direct intramyocardial administration of an adenovirus vector expressing VEGF121 cDNA to individuals with clinically significant severe coronary artery disease. Circulation.

[CR149] Rosenkranz S (2002). Alterations of beta-adrenergic signaling and cardiac hypertrophy in transgenic mice overexpressing TGF-beta(1). Am J Physiol Heart Circ Physiol.

[CR150] Rosenkranz S (2003). beta-adrenoceptor blockade prevents cardiac hypertrophy and failure in transforming growth factor-beta 1 transgenic mice. Eur Heart J.

[CR151] Ross R (1993). Atherosclerosis: current understanding of mechanisms and future strategies in therapy. Transplant Proc.

[CR152] Salomon D (2014). Transforming growth factor beta in cancer: Janus, the two-faced god.. J Natl Cancer Inst.

[CR153] Sandanayake NS (2011). A combination of serum leucine-rich alpha-2-glycoprotein 1, CA19-9 and interleukin-6 differentiate biliary tract cancer from benign biliary strictures. Br J Cancer.

[CR154] Sanderson JE (2001). Transforming growth factor-beta(1) expression in dilated cardiomyopathy. Heart.

[CR155] Sasaki H (1992). Transforming growth factor-beta in the regulation of the immune response. Clin Immunol Immunopathol.

[CR156] Sato Y (1990). Characterization of the activation of latent TGF-beta by co-cultures of endothelial cells and pericytes or smooth muscle cells: a self-regulating system. J Cell Biol.

[CR157] Scheuer J (1982). Physiologic cardiac hypertrophy corrects contractile protein abnormalities associated with pathologic hypertrophy in rats. J Clin Invest.

[CR158] Schiekofer S (2006). Microarray analysis of Akt1 activation in transgenic mouse hearts reveals transcript expression profiles associated with compensatory hypertrophy and failure. Physiol Genomics.

[CR159] Schultz Jel J (2002). TGF-beta1 mediates the hypertrophic cardiomyocyte growth induced by angiotensin II. J Clin Invest.

[CR160] Schumacher B (1998). Induction of neoangiogenesis in ischemic myocardium by human growth factors: first clinical results of a new treatment of coronary heart disease. Circulation.

[CR161] Scott CD, Firth SM (2004). The role of the M6P/IGF-II receptor in cancer: Tumor suppression or garbage disposal?. Horm Metab Res.

[CR162] Seay U (2005). Transforming growth factor-beta-dependent growth inhibition in primary vascular smooth muscle cells is p38-dependent. J Pharmacol Exp Ther.

[CR163] Seeland U (2002). Myocardial fibrosis in transforming growth factor-beta(1) (TGF-beta(1)) transgenic mice is associated with inhibition of interstitial collagenase. Eur J Clin Invest.

[CR164] Serada S (2010). iTRAQ-based proteomic identification of leucine-rich alpha-2 glycoprotein as a novel inflammatory biomarker in autoimmune diseases. Ann Rheum Dis.

[CR165] Serada S (2012). Serum leucine-rich alpha-2 glycoprotein is a disease activity biomarker in ulcerative colitis. Inflamm Bowel Dis.

[CR166] Shi Y, Massague J (2003). Mechanisms of TGF-beta signaling from cell membrane to the nucleus. Cell.

[CR167] Shi ML (2011). Latent TGF-beta structure and activation. Nature.

[CR168] Shou M (1997). Effect of basic fibroblast growth factor on myocardial angiogenesis in dogs with mature collateral vessels. J Am Coll Cardiol.

[CR169] Simons M (2002). Pharmacological treatment of coronary artery disease with recombinant fibroblast growth factor-2: double-blind, randomized, controlled clinical trial. Circulation.

[CR170] Smeets PJ (2008). Transcriptomic analysis of PPARalpha-dependent alterations during cardiac hypertrophy. Physiol Genomics.

[CR171] Stavropoulou A (2010). uPA, uPAR and TGF beta(1) expression during early and late post myocardial infarction period in Rat myocardium. In Vivo.

[CR172] Symes JF (1999). Gene therapy with vascular endothelial growth factor for inoperable coronary artery disease. Ann Thorac Surg.

[CR173] Thannickal VJ (2003). Myofibroblast differentiation by transforming growth factor-beta1 is dependent on cell adhesion and integrin signaling via focal adhesion kinase. J Biol Chem.

[CR174] Thomas DP (2000). Collagen gene expression in rat left ventricle: interactive effect of age and exercise training. J Appl Physiol.

[CR175] Thomas DP (2001). Exercise training attenuates ageing-associated increases in collagen and collagen crosslinking of the left but not the right ventricle in the rat. Eur J Appl Physiol.

[CR176] Thurston G (2002). Complementary actions of VEGF and angiopoietin-1 on blood vessel growth and leakage. J Anat.

[CR177] Tsai S (2009). TGF-beta through Smad3 signaling stimulates vascular smooth muscle cell proliferation and neointimal formation. Am J Physiol Heart Circ Physiol.

[CR178] Udelson JE (2000). Therapeutic angiogenesis with recombinant fibroblast growth factor-2 improves stress and rest myocardial perfusion abnormalities in patients with severe symptomatic chronic coronary artery disease. Circulation.

[CR179] Unger EF (1994). Basic fibroblast growth factor enhances myocardial collateral flow in a canine model. Am J Physiol.

[CR180] Unger EF (2000). Effects of a single intracoronary injection of basic fibroblast growth factor in stable angina pectoris. Am J Cardiol.

[CR181] Vale PR (2001). Randomized, single-blind, placebo-controlled pilot study of catheter-based myocardial gene transfer for therapeutic angiogenesis using left ventricular electromechanical mapping in patients with chronic myocardial ischemia. Circulation.

[CR182] Vallender TW, Lahn BT (2006). Localized methylation in the key regulator gene endothelin-1 is associated with cell type-specific transcriptional silencing. FEBS Lett.

[CR183] van Meeteren LA, ten Dijke P (2012). Regulation of endothelial cell plasticity by TGF-beta. Cell Tissue Res.

[CR184] van Royen N (2002). Exogenous application of transforming growth factor beta 1 stimulates arteriogenesis in the peripheral circulation. FASEB J.

[CR185] Villarreal FJ, Dillmann WH (1992). Cardiac hypertrophy-induced changes in mRNA levels for TGF-beta 1, fibronectin, and collagen. Am J Physiol.

[CR186] Wahl SM (1987). Transforming growth factor type beta induces monocyte chemotaxis and growth factor production. Proc Natl Acad Sci U S A.

[CR187] Wang JS (2011). Ageing-induced collateral dysfunction: impaired responsiveness of collaterals and susceptibility to apoptosis via dysfunctional eNOS signaling. J Cardiovasc Transl Res.

[CR188] Wang X (2013). LRG1 promotes angiogenesis by modulating endothelial TGF-beta signalling. Nature.

[CR189] Watson CJ (2011). Proteomic analysis of coronary sinus serum reveals leucine-rich alpha 2-glycoprotein as a novel biomarker of ventricular dysfunction and heart failure. Circ Heart Fail.

[CR190] Weber KT (1995). Structural remodelling of the heart by fibrous tissue: role of circulating hormones and locally produced peptides.. Eur Heart J.

[CR191] Wen SY (2014). LRG1 is an independent prognostic factor for endometrial carcinoma. Tumor Biol.

[CR192] Wenzel S (2001). Redox-sensitive intermediates mediate angiotensin II-induced p38 MAP kinase activation, AP-1 binding activity, and TGF-beta expression in adult ventricular cardiomyocytes. FASEB J.

[CR193] Wu J (2013). Altered expression of sialylated glycoproteins in ovarian cancer sera using lectin-based ELISA assay and quantitative glycoproteomics analysis. J Proteome Res.

[CR194] Zeisberg EM (2007). Endothelial-to-mesenchymal transition contributes to cardiac fibrosis. Nat Med.

[CR195] Zhang DZ (2002). Efficacy and safety of therapeutic angiogenesis from direct myocardial administration of an adenoviral vector expressing vascular endothelial growth factor 165. Chin Med J.

[CR196] Zhao M (2004). Microarray analysis of gene expression after transverse aortic constriction in mice. Physiol Genomics.

[CR197] Zolk O (2000). Decreased expression of the cardiac LIM domain protein MLP in chronic human heart failure. Circulation.

